# A Nomadic Infrastructure with Hierarchical Block Tracking and Surveillance Resolution in Satellite Networks

**DOI:** 10.3390/s26010180

**Published:** 2025-12-26

**Authors:** Minsoo Kim, Jalel Ben-Othman, Lynda Mokdad, Paolo Bellavista, Hyunbum Kim

**Affiliations:** 1 Department of Embedded Systems Engineering, Incheon National University, Incheon 22012, Republic of Korea; rlaalstn19@inu.ac.kr; 2 LISSI Lab, University of Paris-Est, 40514 Créteil, France; jalel.ben-othman@u-pec.fr; 3 LACL Laboratory, Department of Computer Science, University of Paris-Est, 94010 Créteil, France; lynda.mokdad@u-pec.fr; 4 Department of Computer Science and Engineering, University of Bologna, 40136 Bologna, Italy; paolo.bellavista@unibo.it

**Keywords:** block tracking, sensing, nomadic, satellite, surveillance, communication

## Abstract

In this paper, we propose a multi-layered hierarchical block tracking (HBT) system for continuous real-time sensing and efficient network management of highly mobile nomadic infrastructure. In order to solve the limitations of the existing high-resolution satellite direct connection method in the fast tracking and management of mobile infrastructure due to the high processing delay and large data processing burden, we introduce event-based data abstraction and Infra Map management in a multi-layered network consisting of a Low Earth Orbit satellite (LEO), high-altitude platform (HAP), and low-altitude platform (LAP). Through this, unnecessary data transmission is minimized and processing speed and Surveillance Resolution (SR) are improved. Experimental results show that the HBT structure achieves the improved SR at low-speed conditions, maintaining high tracking stability even under dynamic movement scenarios, while reducing processing delay when compared to the existing LEO–ground direct communication. As a result, we verify that the HBT structure shows lower processing delay and high tracking stability when compared to the existing LEO–ground direct communication.

## 1. Introduction

Traditional network infrastructure relies heavily on static components such as base stations, optical backbones, and ground stations to ensure stable transmission from fixed locations [1,2,3,4,5]. However, the rapid proliferation of autonomous vehicles, drones, and maritime logistics systems has exposed the constraints of this conventional model, which was not originally designed to support highly mobile entities requiring continuous connectivity [6,7,8]. To address these challenges, we introduce the concept of *nomadic infrastructure*. Unlike traditional mobile terminals that act merely as end-users, nomadic infrastructure extends the network boundary by enabling moving entities to actively participate in sensing and connectivity. Consequently, the network topology must be managed as a dynamic, time-varying graph rather than a static cell layout.

A fundamental challenge arises when nomadic infrastructure relies on satellite connectivity, particularly in remote environments such as oceans or deserts where terrestrial support is absent. Existing approaches primarily utilize direct LEO-to-ground communication. However, this architecture faces systemic limitations when applied to highly mobile infrastructure. First, direct connections suffer from cumulative delays due to propagation, handover overhead, and heavy image processing loads, often exceeding hundreds of milliseconds [9,10,11,12,13]. Second, high-frequency satellite signals are susceptible to atmospheric attenuation from rain and clouds, leading to link instability. Third, transmitting raw sensor data or high-resolution images for every event consumes excessive bandwidth and energy, which is critical for battery-powered nomadic nodes. These factors necessitate an architectural shift from direct raw data transmission to a hierarchical, event-driven approach.

In this paper, we propose a multi-layered network model known as *hierarchical block tracking (HBT)* to facilitate seamless and efficient connectivity between nomadic infrastructure and satellites. Unlike the direct connection method, HBT leverages a satellite–high-altitude platform (HAP)–low-altitude platform (LAP) hierarchy to manage infrastructure in “blocks”. By employing event-driven data abstraction—transmitting only lightweight updates instead of raw imagery—and separating the control plane (Infra Map) from the data plane, HBT significantly reduces processing delay and enhances tracking continuity.

The main contributions of this paper are summarized as follows:

- We define *nomadic infrastructure* as an integrated network component and analyze the limitations of direct satellite coupling. We then propose the HBT architecture tailored to the mobility and scalability needs of such infrastructure, aiming to provide an optimized reference for autonomous logistics and aviation communications.
- We introduce an event-driven *HBT* mechanism that mitigates high latency and signal attenuation. By utilizing a multi-layered structure (LEO-HAP-LAP) and data abstraction, the proposed system minimizes unnecessary data transmission and optimizes communication pathways compared to direct LEO–ground links.
- We propose *Surveillance Resolution (SR)* as a novel metric to assess continuous tracking performance. Unlike instantaneous coverage probability, SR quantifies the temporal continuity of tracking. We verify that the HBT structure achieves higher tracking stability and lower processing delay through extensive comparative simulations.

In our evaluation, the proposed HBT-based architecture reduces the normalized processing delay by up to roughly 60–80% compared with a direct LEO–terrestrial baseline under ultra-high-resolution imaging with low-to-moderate event probabilities. Furthermore, it improves SR at low speeds from about 56–58% for the baseline to approximately 88%, while maintaining more than 70% tracking continuity across most speeds once the LAP sensing radius is sufficiently enlarged.

The remaining parts of this paper are organized as follows. Section 2 reviews related work. Section 3 presents the system overview, system settings, problem definition, and the metrics used for evaluation. Section 4 details the proposed HBT algorithms and the Infra Map management scheme. Section 5 evaluates the performance of the proposed methods through extensive simulations. Finally, Section 6 discusses future research directions, and Section 7 concludes this paper.

## 2. Related Work

### 2.1. Satellite Network Architectures and Data Abstraction

Early satellite connectivity relied on direct LEO-to-ground communication, which suffers from significant cumulative latency due to propagation and processing delays [14,15,16]. Even hybrid GEO-LEO architectures cannot fully eliminate these delays or the throughput constraints inherent in sporadic, low-rate sensor traffic [17]. To address this, multi-tier architectures integrating UAVs and edge computing (SatEC) have emerged to enable task offloading and distributed processing [18,19]. Hierarchical reinforcement learning has also been explored for task scheduling across space–air–ground networks [20].

However, these existing hierarchical systems typically treat tasks as atomic units requiring full data uploads. They lack a mechanism for *block-level state management* or *event-driven abstraction*, meaning raw sensor data is transmitted in its entirety rather than being filtered at lower layers. This limitation prevents efficient synchronization for highly mobile infrastructure, motivating our proposed HBT architecture, which transmits only compacted delta updates.

### 2.2. Mobility Management in Non-Terrestrial Networks

Tracking mobile infrastructure presents scalability challenges due to the rapid orbital velocity of LEO satellites (approx. 7.6 km/s), which necessitates frequent handovers [21]. While recent studies have applied Deep Q-learning and XGBoost to optimize handover decisions [22,23], these approaches primarily focus on maintaining link quality during beam switching rather than ensuring the continuous semantic tracking of the infrastructure itself. Furthermore, insufficient constellation density often leads to coverage gaps and service discontinuities, which are critical failures for real-time applications such as autonomous vehicle monitoring [24].

### 2.3. Performance Metrics for Continuous Surveillance

Performance evaluation in satellite networks has traditionally relied on standard QoS metrics (throughput, delay, and packet loss) [25,26] or instantaneous coverage probability based on SNR thresholds [27,28,29,30]. However, these metrics capture coverage as a binary or probabilistic state at discrete time points. They fail to quantify the *temporal dimension* of surveillance—specifically, how continuously a system can track a moving target over an extended period. To address this gap, we introduce *Surveillance Resolution (SR)*, a novel metric designed to measure the persistence of tracking continuity, distinguishing our work from prior snapshot-based evaluations.

## 3. Proposed System

### 3.1. System Overview

We consider a three-tier architecture comprising LEO satellites, HAPs, and LAPs to sustain continuous connectivity for nomadic infrastructure while minimizing dependence on direct satellite-to-ground links. As illustrated in the architecture, LEO satellites maintain a global view and manage coordination across blocks; HAPs supervise groups of LAPs within predefined blocks and handle aggregation of local updates; and LAPs perform sensing near targets to produce compact status reports.

Responsibilities are explicitly separated into a *control plane* and a *data plane*. The control plane maintains a block-level Infra Map, while the data plane handles sensing, pre-processing, and dissemination. Updates follow an event-driven policy: a typical cycle begins with sensing at LAPs, continues with aggregation at HAPs, proceeds to a global map update at the satellite, and concludes with dissemination to affected blocks.

**Figure 1:**
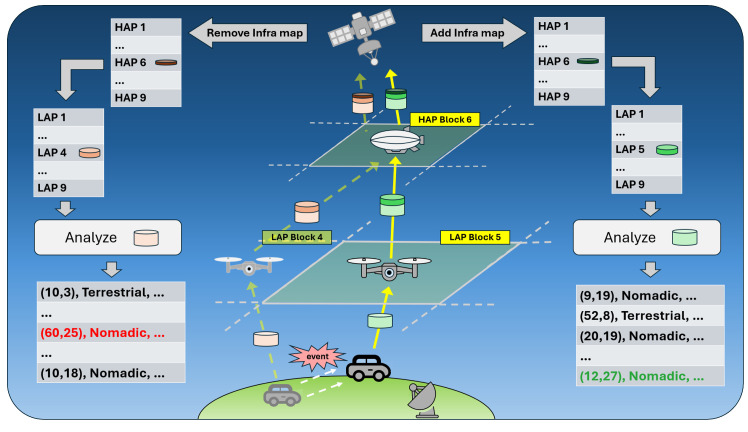
Overview of the HBT architecture with event-driven monitoring and Infra Map updates.

Compared to prior systems, our architecture introduces three distinctive elements: (1) the satellite tracks block-level states rather than individual units; (2) HAPs act as relays for event-driven updates only; and (3) LAPs locally decide which events warrant reporting. This approach fundamentally shifts where information is generated and consumed. Table 1 summarizes the qualitative differences between the direct LEO baseline and the proposed HBT structure. Figure 1 presents an overview of the HBT architecture and Infra Map updates.

On top of this architecture, the LEO periodically runs a lightweight reassignment controller. This controller inspects block-level indicators from the Infra Map and reallocates LAP coverage when stress conditions are met. The concrete trigger conditions and assignment cost function are defined in Section 4.2 along with Algorithm 1.**Algorithm 1** LAP—Delta Detect and Encode.1:**Class** LAP:2:    **Initialize** local_cache $S_{l}\leftarrow ⌀$, seq ←03:    **Params** (θ,cooldown,rate_cap)4:**Function** ProcessObservation($S_{l}\left(t\right)$, ts):5:     $diff\leftarrow \mathrm{ComputeDiff}(S_{l}\left(t\right),\;\mathrm{prev}=S_{l}\left(t\;-\;1\right))$6:     **if** diff≥θ **and** CooldownOK(ts) **then**7:         seq←seq+18:         Δ←EncodeDelta(type,infra_id,block_id,value,ts,seq,crc)9:         **if** RateOK(rate_cap) **then**10:           PushToQueue($Q_{LH},\Delta$)11:       **end if**12:   **end if**13:   UpdateCache($S_{l}\left(t\right)$)14:   **return**

### 3.2. Experimental Testbed and System Parameters

To validate the feasibility and performance of the proposed HBT architecture, we constructed a *sensor-based experimental testbed* using a discrete-event simulation framework. This testbed explicitly models the dynamic interaction between the hierarchical network layers (LEO, HAP, and LAP) and the mobile nomadic infrastructure within a continuous sensing environment. The specific topology, sensor specifications, and simulation parameters used in this testbed are defined as follows:

- **Spatio-temporal discretization**: The surveillance area is modeled as a 100×100 2D grid. The simulation time step is Δt=1 slot and the horizon is T=300 slots.
- **Topology**: One LEO manages nine HAPs, and each HAP manages nine LAPs (1:9:81), providing a balanced trade-off between coverage symmetry and management overhead.
- **LEO/HBT geometry**: Four LEO footprints are centered at (50, 10), (50, 90), (10, 50), and (90, 50) with radius $R_{\mathrm{LEO}}=27$ cells. LAPs are placed on a regular grid with six-cell spacing over ${\left[1,99\right]}^{2}$ with a sensing radius of $R_{\mathrm{LAP}}=3$ cells. The LEO is visible for $T_{\mathrm{vis}}=13$ slots every $T_{\mathrm{orb}}=20$ slots.
- **Entity load**: Each LAP monitors $N_{\mathrm{infra}}\in \left\{10,50\right\}$ infrastructure units in SR experiments and up to 100 units in processing-delay experiments.
- **Abstraction payload**: A per-infrastructure abstracted string (location, speed, status, etc.) of size ≈ 8 KB is generated at the LAP.
- **Event dynamics**: The event probability *p* takes values in {0.1,0.3,0.5,0.7,0.9,1.0}.
- **Processing rate**: It is normalized to 30 KB/ms (≈240 Mb/s), corresponding to a mid-range GPU-assisted pipeline.
- **Power profile**: LAPs operate in a low-duty-cycle sensing mode, uploading only event-driven payloads. This reduces radio and processing energy by orders of magnitude compared to continuous streaming, which is critical for battery-powered platforms.

Unless otherwise stated, all numerical results assume this balanced 1:9:81 hierarchy.

### 3.3. Problem Definition

We formulate the tracking of nomadic infrastructure as a joint optimization problem over *processing delay* and *Surveillance Resolution (SR)*. Unlike traditional studies that evaluate instantaneous coverage or routing capacity, our framework explicitly couples (i) the end-to-end delay for event acquisition and (ii) the temporal continuity of observation.

The operational view consists of an *update phase*, where local sensing propagates abstracted changes upward, and a *deployment phase*, where the refreshed global state guides resource reassignment. We treat processing delay and SR not just as outcomes but as design criteria. For a system configuration *x* and architecture a∈{Direct,HBT}, let $D_{a}\left(x\right)$ and ${\mathrm{SR}}_{a}\left(x\right)$ denote the expected normalized delay and SR. A network operator can prioritize fast reaction ($P_{1}$) or tracking continuity ($P_{2}$):

(1) $$P_{1}\left(a\right):\;\min_{x}D_{a}\left(x\right)\;s.t.\;{\mathrm{SR}}_{a}\left(x\right)\geq {\mathrm{SR}}_{\min},$$

(2) $$P_{2}\left(a\right):\;\max_{x}{\mathrm{SR}}_{a}\left(x\right)\;s.t.\;D_{a}\left(x\right)\leq D_{\max}.$$

These metrics define the objectives for tuning architectural parameters such as LAP density and abstraction thresholds.

### 3.4. Metrics

#### 3.4.1. Processing Delay

Processing delay defines the end-to-end system reaction time—from event detection at the edge to global state availability. Based on measurements from commercial LEO systems (e.g., Starlink RTT ≈ 30–80 ms [31,32]), we normalize our baseline direct LEO–terrestrial delay to 100 (representing ≈ 50 ms).

We define the effective event arrival rate (Table 2) $\lambda^{\mathrm{LEO}}$ and offered traffic $\Gamma^{\mathrm{LEO}}$ reaching the satellite as follows:

(3) $$\lambda^{\mathrm{LEO}}=\frac{m\;r}{\Delta t}\;p\;N_{b}\;B,$$

(4) $$\Gamma^{\mathrm{LEO}}=s_{\Delta }\;\lambda^{\mathrm{LEO}}.$$

Here, *m* and *r* represent the merge (deduplication) and rate limit factors at the HAP, respectively.

The theoretical transmission lower bounds for the direct baseline ($D_{\mathrm{LEO}}$) and the two-hop HBT architecture ($D_{\mathrm{HBT}}$) are modeled as followed:

(5) $$D_{\mathrm{LEO}}=\frac{s_{\mathrm{img}}}{R_{\mathrm{LT}}},$$

(6) $$D_{\mathrm{HBT}}=\frac{s_{\Delta }}{R_{\mathrm{HL}}}+\frac{s_{\Delta }}{R_{\mathrm{LH}}}.$$

Equation (6) demonstrates that HBT reduces delay by transmitting small abstracted payloads ($s_{\Delta }$) despite the multi-hop path, whereas the direct baseline (Equation (5)) is constrained by large image sizes ($s_{\mathrm{img}}$). Physical layer impairments are abstracted into effective link rates; weather impacts are discussed in Section 6.1.

#### 3.4.2. Surveillance Resolution

Continuous tracking is essential for nomadic infrastructure. Existing metrics like coverage probability or Age of Information (AoI) often fail to capture the *temporal continuity* of tracking. We introduce *Surveillance Resolution (SR)* to measure the fraction of time an entity remains effectively monitored over a horizon *T* (Table 3).

Formally, the per-slot coverage flag is defined as follows:

(7) $${\mathrm{cov}}_{i}\left(t\right)=\left\{\begin{array}{cc} 1, & d_{i}\left(t\right)\leq R\\ 0, & \mathrm{otherwise}. \end{array}\right.$$

The individual and system-level SRs are then derived as follows:

(8) $${\mathrm{SR}}_{i}=\frac{1}{T}\sum_{t=1}^{T}{\mathrm{cov}}_{i}\left(t\right),$$

(9) $$\mathrm{SR}=\frac{1}{\left|I\right|}\sum_{i\in I}{\mathrm{SR}}_{i}.$$

**Figure 2:**
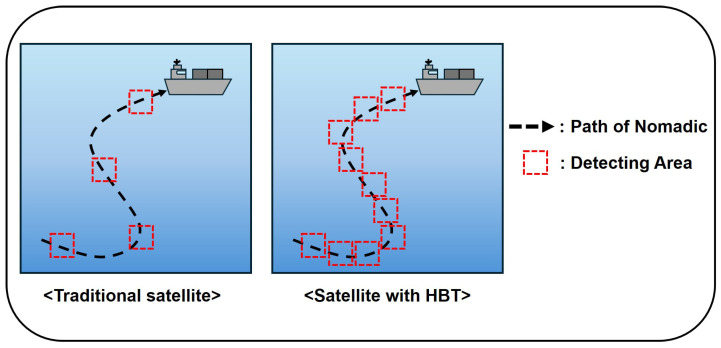
The concept of SR: Comparison between traditional satellite communication’s intermittent coverage (low SR) and HBT’s continuous tracking (high SR).

For instance, if a target is tracked for 8 minutes out of a 10 min trajectory, the SR is 80%. Figure 2 illustrates this concept. In the direct LEO baseline, ${\mathrm{cov}}_{i}\left(t\right)$ frequently drops to 0 when the satellite moves out of view or signal attenuation occurs. In contrast, HBT maintains higher SR by utilizing the LAP-HAP layer to perform handovers and maintain block-level tracking ($d_{i}\left(t\right)\leq R_{\mathrm{LAP}}$) even when the LEO is not directly visible.

## 4. Proposed Schemes

In this section, we detail the operational algorithms of the HBT architecture: *LAP—Delta Detect and Encode*; *HAP—Merge, Batch, and Gate*; and the *Infra Map* update protocols. These schemes constitute the core logic implemented within the *experimental testbed described in Section 3.2*. By defining these interaction protocols, we establish a logical bridge between the system model (Section 3) and the performance evaluation (Section 5).

The following algorithms instantiate the operational core of the proposed HBT-based architecture. Algorithm 1 specifies the LAP-side delta detection and encoding procedure, which decides when local state changes are significant enough to be reported upward and thus controls event generation and the amount of uplink traffic. Algorithm 2 describes the HAP-side merge–batch–gate mechanism that aggregates LAP events at the block level and enforces rate caps toward the LEO layer, providing scalable admission control under varying infrastructure densities and event probabilities. Algorithm 3 captures the LEO-level reassignment controller, which periodically inspects block-level indicators stored in the Infra Map and adapts LAP coverage when stress conditions are met, thereby linking the problem formulation in terms of processing delay and SR to concrete control actions. Together, these three algorithms implement the event-driven abstraction, traffic shaping, and block-level adaptation mechanisms that distinguish the proposed HBT architecture from the direct LEO–terrestrial baseline.

### 4.1. Hierarchical Block Tracking

Nomadic infrastructure aims to provide a more efficient network management structure rather than simply relying on direct satellite connections. Traditional mobile infrastructure is based on fixed base stations, providing a stable network environment. However, since nomadic infrastructure is highly mobile, its network topology constantly changes. As a result, it is inefficient for satellites to track individually and manage all mobile infrastructure, leading to increased network complexity. To address this issue, we introduce the concept of HBT and propose a network management approach utilizing the Infra Map to enhance efficiency. HBT does not require satellites to track mobile infrastructure directly. Instead, it employs LAP and HAP layers to manage the network in block units. This structure enables satellites to effectively manage broader areas while reducing complexity associated with the mobility of individual infrastructure elements. HBT leverages a satellite–HAP-LAP hierarchy to manage the network in block units, enhancing network stability and operational efficiency compared to traditional direct satellite-to-ground connections.

**Figure 3:**
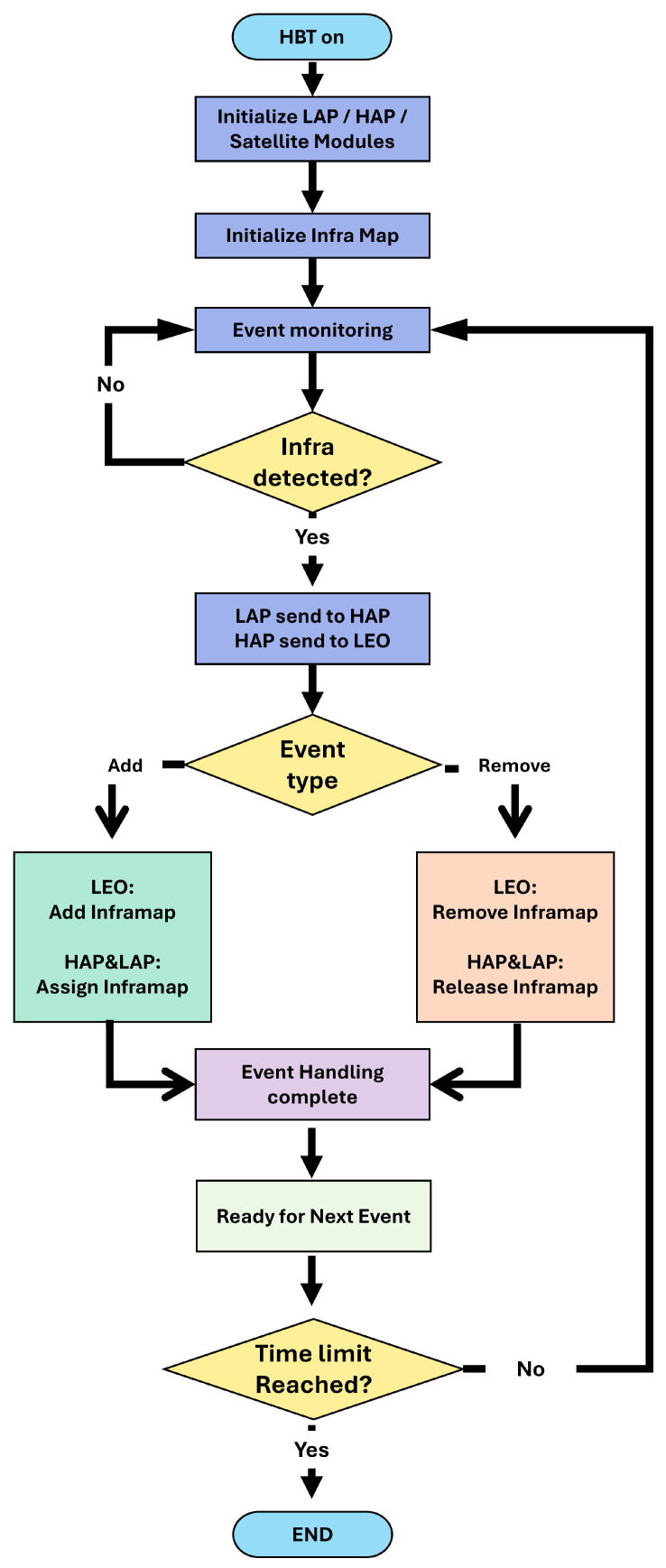
HBT flowchart illustrating the continuous control loop for event-driven monitoring and Infra Map updates, including the explicit termination condition.

The overall operation of HBT follows an event-driven process, as shown in Figure 3. *This pipeline is formalized by two procedures: Algorithm 1 (LAP—Delta Detect and Encode) and Algorithm 2 (HAP—Merge, Batch, and Gate).* Upon detecting infrastructure changes, LAP modules escalate events to the HAP, which further notify LEO to update the Infra Map. *In Algorithm 1*, a LAP triggers when a local change exceeds a threshold θ after a cooldown window, encodes a compact delta record (type, key, value, timestamp, monotonic sequence, ans checksum), enforces a local rate cap to tame bursts, and pushes the delta to the LAP → HAP queue while updating its cache. In Algorithm 2, the HAP aggregates deltas over a batching window $T_{g}$, merges duplicates by key under a priority rule, and admits the batch to the HAP → LEO link using a token-bucket limiter with cap $r_{\max}$. The HAP logs the *merge factor m* and the *acceptance factor r*, which feed the offered-load model in Equations (1) and (2). This hierarchical event handling allows satellites to efficiently manage add/remove operations of infrastructure states in block units, minimizing unnecessary data transmission while ensuring real-time adaptability to dynamic environments.
**Algorithm 2** HAP—Merge, Batch, and Gate (upstream).1:**Class** HAP:2:     **Params** $\left(T_{g},\;r\_max\right)$3:     **MergeKey** ← (block_id, key)4:     **PriorityRule**: *Add/Remove* ≻*Update*, newer ts first5:     **Counters** $n_{in}\leftarrow 0$, $n_{out}\leftarrow 0$6:**Function** MergeBatchGate():7:     Batch←⌀8:     **while** WithinWindow($T_{g}$) **do**9:         $\Delta \leftarrow \mathrm{PopQueue}(Q_{LH})$ **if available**10:       **if** Δ **then**11:           Batch←Batch∪{Δ}12:           $n_{in}\;\leftarrow \;n_{in}\;+\;1$13:       **end if**14:   **end while**15:   $Batch^{\prime }\leftarrow \mathrm{MergeByKey}\left(Batch,\;\mathrm{MergeKey}\right)$16:   $Batch^{\prime \prime }\leftarrow \mathrm{SortByPriority}\left(Batch^{\prime },\;\mathrm{PriorityRule}\right)$17:   **for** $\Delta \in Batch^{\prime \prime }$ **do**18:       **if** Admit(r_max) **then**19:          PushToQueue($Q_{HL},\Delta$)20:          $n_{out}\;\leftarrow \;n_{out}\;+\;1$21:       **else** /* non-admitted deltas are dropped at HAP */22:       **end if**23:   **end for**24:   $m\leftarrow \frac{|Batch^{\prime }|}{\max(1,|Batch|)}$25:   $r\leftarrow \frac{n_{out}}{\max(1,|Batch^{\prime }|)}$26:   **return** $\left(Batch^{\prime \prime },m,r\right)$

To make the upstream behavior of Algorithm 2 reproducible, we explicitly define the priority rule and the admission gate. Each delta Δ produced by Algorithm 1 carries an operation type *type* ∈ {Add, Remove,Update}, a block identifier, a key, and a timestamp (or monotonically increasing sequence number). The function MergeByKey(Batch,MergeKey) first coalesces multiple deltas for the same Infra Map entry, identified by MergeKey=(block_id,key). The resulting set $Batch^{\prime }$ is then ordered by PriorityRule: structural changes (Add/Remove) are ranked ahead of value changes (Update), and within the same type more recent timestamps are placed first. The function $\mathrm{Admit}(r_{\max})$ implements a token-bucket limiter per batching window of length $T_{g}$: at the beginning of each window, at most $r_{\max}$ tokens are available, and each admitted delta consumes one token. When the bucket is empty, Admit(·) returns *false* and the remaining deltas in $Batch^{\prime \prime }$ are simply dropped at the HAP rather than buffered or recompressed. Consequently, the acceptance factor *r* in Algorithm 2 is exactly the fraction of merged deltas that pass this gate. Because Infra Map updates at the LEO layer are idempotent and resolved with a latest-wins rule, dropping low-priority deltas only coarsens the temporal resolution of state changes without breaking eventual consistency; more sophisticated buffering and loss recovery strategies are left for future work.

The concrete parameter settings used in Algorithms 1 and 2 are as follows. Each infrastructure state is normalized to the range [0,1], and the state change threshold is set to θ=0.05, meaning that a delta is generated only when the normalized state changes by more than 5%. To avoid excessive event bursts, we impose a per-infrastructure cooldown window of five time slots, so that the same infrastructure cannot emit more than one delta within five consecutive slots even if the threshold is repeatedly exceeded. At the LAP/HAP interface, a per-LAP rate cap of 10 abstraction messages per slot is applied, which limits the number of event-driven updates forwarded to the upper layer and prevents saturation of the HAP–LEO uplink while still capturing the dominant state changes. Unless otherwise stated, all simulation results are obtained with these parameter values.

The core concept of HBT is that satellites do not directly track individual nomadic infrastructure but instead utilize the LAP and HAP as intermediary layers, managing the network in block units. In conventional methods, satellites had to individually recognize and track each nomadic infrastructure, leading to an exponential increase in computational load as the network scaled. However, in the HBT model, a single satellite manages nine HAPs, and each HAP oversees nine LAPs, forming a hierarchical structure. This configuration of assigning nine HAPs per LEO and nine LAPs per HAP was determined to balance spatial granularity and system efficiency. A 3×3 block structure provides symmetry in coverage and simplifies hierarchical management across layers, enabling efficient event detection and minimizing processing overhead. While alternative configurations could be considered, nine units were identified as an optimal trade-off that maintains tracking stability without introducing excessive communication or computational load. This design allows a single satellite to track up to 81 LAP regions in block units. Through this approach, satellites primarily oversee the LAP-HAP network, while the detailed management of nomadic infrastructure is handled at the LAP and HAP levels, transmitting only the necessary information to the satellite. Table 1 specifies a comparison between direct LEO–terrestrial communication and the HBT architecture.

It is worth noting that the proposed HBT formulation is not tied to the specific 1:9:81 hierarchy. Alternative fan-out patterns, such as 1:4:16 or 1:16:256, can be modeled by using the same block-level update and forwarding rules while changing only the number of blocks managed at each layer. In such cases, the absolute values of processing delay and SR are scaled by the resulting block density and coverage area, but the qualitative trend—that the HBT architecture reduces processing delay and enhances SR compared to the direct LEO–terrestrial baseline—remains unchanged. Therefore, the 1:9:81 topology is adopted as a representative balanced case rather than a configuration specifically tuned to a particular scenario.

### 4.2. Infra Map and Abstraction

If satellites collect and process all information directly whenever nomadic infrastructure changes in real time, the computational burden of satellites will increase rapidly and unnecessary data traffic will occur, which will likely reduce the efficiency of network operation. To handle this issue, we devise a method of efficiently managing the network state of nomadic infrastructure and minimizing the data processing burden of satellites by introducing the concepts of *Infra Map* and *abstraction*.

The Infra Map is a set of information designed to intuitively visualize and manage the deployment of nomadic and terrestrial infrastructure in an HBT-based network. Rather than directly tracking the nomadic infrastructure, satellites can reduce the operating burden of the network by monitoring the network status based on the Infra Map and updating the location information of objects only when necessary. The Infra Map performs three main functions in network operation. First, through the network visualization function, the distribution of nomadic and terrestrial infrastructure can be grasped at a glance. Second, satellites can optimize the network based on the data filtered by the LAP and HAP, thereby minimizing unnecessary operations. Third, it supports updating the network in real time by reflecting the information changed in each block unit.

In order to create such an Infra Map, it is necessary to efficiently organize the data collected by the LAP and HAP. Then, *abstraction* is a data management technique that simplifies the original data collected by the LAP and HAP so that satellites can quickly process only the necessary key information. Network operation is much more efficient if the satellite does not directly analyze the original image or a large amount of infrastructure data and only the core information is coded and managed. For example, if a specific nomadic infrastructure exists in HAP 1 and LAP 7, it can be expressed as H1L7N (HAP 1, LAP 7, nomadic infrastructure), and if a specific terrestrial infrastructure exists in HAP 3 and LAP 9, it can be expressed as H3L9T (HAP 3, LAP 9, terrestrial infrastructure). Simplifying network information in this way allows satellites to quickly grasp the current state of the network without having to directly process the original data, and based on this, they can continuously update Infra Maps.

Algorithm 3 formalizes the LEO-side reassignment controller that consumes these abstracted deltas and adjusts LAP-to-block assignments based on block-level indicators. To keep the policy simple and reproducible, we use two scalar metrics per block *b*: the sliding-window SR $SR_{b}\left(t\right)$ and the average processing delay $Delay_{b}\left(t\right)$. We choose fixed thresholds $SR_{\min}=0.8$ and $Delay_{\max}$ = 30 ms and a minimum hold time $T_{\mathrm{hold}}=10$ control periods. The TriggerOn(*Triggers*, *Stats*) function in Algorithm 3 returns 1 if there exists at least one block *b* such that $SR_{b}\left(t\right)<SR_{\min}$ and $Delay_{b}\left(t\right)>Delay_{\max}$ and the elapsed time since the last reassignment exceeds $T_{\mathrm{hold}}$; otherwise, it returns 0. Thus, reassignment is invoked only when a block is simultaneously under-covered and congested for a sufficiently long period.
**Algorithm 3** LEO—Reassign (Trigger and Assignment Policy).1:**Class** LEO:2:      **Inputs** updated map $M_{t}$3:      **Params Triggers**(demand, SR, delay, queue), min_hold, **CostFn**4:**Function** Reassign():5:      **if** HoldTimerNotExpired(min_hold) **then return**6:      Stats ← ComputeIndicators($M_{t}$)  7:      **if** TriggerOn(**Triggers**, Stats) **then**8:          Req← RankBlocksByDemand($M_{t}$)9:          Avail← AvailableLAPs($M_{t}$)10:        **for** b∈Req **do**11:           $lap^{★}\leftarrow \arg\min_{l\in Avail}\mathrm{CostFn}\left(l,b\right)$12:           Assign$\left(lap^{★}\to b\right)$; Update(Avail)13:        **end for**14:        BroadcastDownlink(Assign/Release commands)15:        ResetHoldTimer()16:    **end if**17:    **return**

To select a new LAP when reassignment is triggered, the controller evaluates a simple assignment cost for each candidate LAP–block pair (*ℓ*, *b*). Let *d*(*ℓ*, *b*) denote the Euclidean distance between LAP *ℓ* and the geometric center of block *b*, and let *L*_*ℓ*_ be the number of blocks currently served by *ℓ*. We then define

(10) $$\mathrm{CostFn}\left(l,b\right)=\alpha_{\mathrm{dist}}\;d\left(l,b\right)+\alpha_{\mathrm{load}}\;L_{l},$$

and Algorithm 3 selects the LAP with the minimum cost among the available candidates. In the reported simulations we set $\alpha_{\mathrm{dist}}=1$ and $\alpha_{\mathrm{load}}=5$, so that the controller primarily prefers nearby LAPs while still avoiding excessive load on a single LAP. When several LAPs have the same minimum cost, the controller keeps the current assignment to prevent unnecessary switching.

On the LEO side, admitted deltas from the HAP are applied to the Infra Map using an atomic update procedure (Algorithm 3). The satellite verifies timestamps and sequence numbers, resolves conflicts with a latest-wins rule, applies add/remove/update operations idempotently, and bumps the map version. The resulting compact, versioned map is then consumed by the reassignment controller defined above and implemented in Algorithm 3, keeping satellite compute bounded and focused on control.

In addition, in order to efficiently synchronize network information during the operation of Infra Map and abstraction, this study introduces an event-driven update. The LAP and HAP adopt a method of updating the Infra Map only when the location of the nomadic or terrestrial Infrastructure changes, rather than periodically transmitting the collected data to the satellite. This increases the efficiency of data transmission by delivering only the key information required by the satellite while maintaining the real-time performance of the network. In addition, the event-driven update method enables immediate reflection when network changes occur, enabling stable operation of the network even if the nomadic infrastructure moves. This method maximizes the management efficiency of the network by reducing unnecessary data traffic and ensuring that the Infra Map is always up to date.

By the LAP and HAP managing network information in real time, satellites perform optimized network operations according to the Infra Map, and the data processing burden of satellites can be reduced and continuous connectivity of nomadic infrastructure can be guaranteed. In addition, flexible network operation that can reflect the network state in real time while reducing unnecessary data traffic is possible through event-driven updates. Next, to verify how effective the network model proposed in this study is in practice, we will explain how to evaluate the continuous tracking performance of nomadic infrastructure by introducing SR.

### 4.3. Complexity Analysis

To demonstrate the feasibility of the proposed HBT architecture compared to the direct LEO–terrestrial baseline, we analyze the computational complexity of the algorithms. Let $N_{infra}$ be the number of infrastructures per LAP, and let the satellite image resolution be H×W pixels.

In the baseline direct communication, the satellite or ground station must process the entire high-resolution image to detect objects. Standard object detection algorithms typically require operations proportional to the number of pixels or feature map grids. Thus, the baseline complexity is approximately O(H·W), which imposes a heavy computational burden and high processing delay, as shown in the experimental results.

In contrast, the proposed HBT operates on discrete events. The complexity breakdown is as follows:

(1) **LAP Layer (Algorithm 1):** The LAP checks the state difference for each monitored infrastructure. Since this involves simple arithmetic operations for $N_{infra}$ units, the complexity is $O(N_{infra})$. This linear complexity is lightweight enough for battery-powered LAP devices.
(2) **HAP Layer (Algorithm 2):** The HAP aggregates *M* delta messages received from its child LAPs. The dominant operations are deduplication and priority sorting. Using a hash map for merging takes expected O(M), and sorting the merged batch takes O(MlogM). Since *M* represents only the subset of infrastructures that moved, M≪H·W.
(3) **LEO Layer (Algorithm 3):** The satellite reassignment controller manages *B* blocks and *L* available LAPs. The cost calculation and greedy assignment in Algorithm 3 involve iterating through blocks and finding the best LAP, resulting in a complexity of O(B·L). Given that *B* and *L* are structural constants (e.g., 81 units) and significantly smaller than the number of raw data pixels, the overhead is negligible.

Consequently, the total system complexity shifts from the pixel domain O(H·W) to the event domain $O(N_{infra}+M\log M)$, theoretically justifying the reduced processing delay and energy efficiency of the HBT architecture.

## 5. Evaluation

In this section, we evaluate the performance of the proposed schemes using the *experimental testbed and parameters defined in Section 3.2*. The simulation executes the event-driven algorithms detailed in Section 4 to measure quantitative metrics such as processing delay and SR under varying infrastructure densities and event probabilities.

### 5.1. Comparison of Processing Delay Considering Spatial Resolution and Event Dynamics

This experiment quantitatively compares the existing LEO–terrestrial and proposed HBT structures, focusing on processing delay, one of the key performance indicators for monitoring system design in a highly mobile infrastructure environment.

The traditional LEO–terrestrial structure is a method of receiving high-resolution satellite images at regular intervals, comprehensively monitoring the entire area, and collectively processing them centrally. This has the advantage of being able to secure information on all infrastructures in real time, but it is inefficient because the same processing load is generated even in static areas where there is no change. On the other hand, the proposed HBT structure follows an event-based communication structure that processes the state of the infrastructure in the form of abstracted strings through the abstraction process at the LAP unit and transmits data to higher layers such as the HAP and satellite only when changes are detected. At this time, the concept of event probability was introduced to model the characteristics of event-based communication. Event probability refers to the proportion of infrastructure that has actually moved or changed in state among the infrastructure areas that the LAP is in charge of.

In the traditional LEO–terrestrial structure, satellite image resolution was modeled in three ways. A 132.5 MB ultra-high-resolution image containing 15 cm information per pixel, 24.2 MB high-resolution image of 30 cm per pixel, image, and 50 MB virtual resolution image that is judged to be able to identify infrastructure units were also included in the experiment. It was assumed that all images were transmitted and processed through direct communication between LEO and the ground.

**Figure 4:**
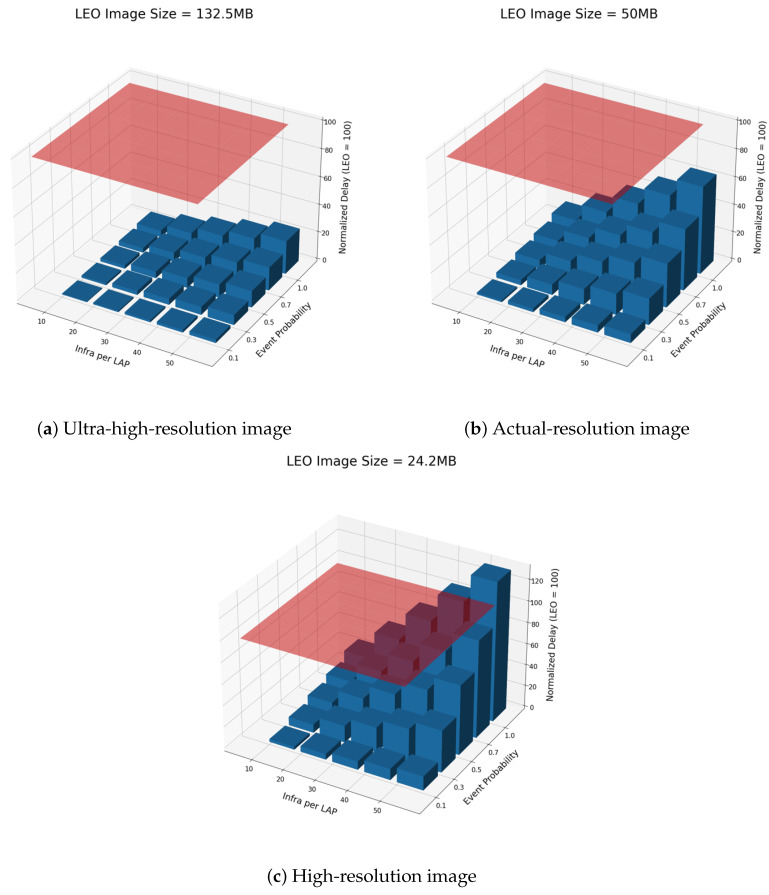
Processing delay comparison between the direct LEO–terrestrial baseline (red plane, normalized to 100) and the proposed HBT architecture as the satellite image size, the number of infrastructures per LAP, and the event probability vary.

The resolution of satellite images was identified as a variable that significantly affects the performance difference between HBT and LEO structures through Figure 4, the result of the processing delay comparison experiment. First of all, in the 132.5 MB ultra-high-resolution image condition containing 15 cm of information per pixel, the HBT structure consistently recorded a lower processing delay than the LEO structure in the combination of all infrastructure counts and event occurrence probabilities. This is because the LEO structure cannot avoid an increase in processing time due to the structural characteristics of transmitting and processing a large amount of the entire image every time, while HBT was able to maintain relatively high efficiency because it selectively processes only the infrastructure in which changes have occurred. Next, in the 50 MB virtual resolution condition, even if the number of infrastructures increases, if the probability of event occurrence is low, such as 0.1 0.3, the performance advantage of HBT is maintained, which is interpreted as a positive signal in terms of system scalability. However, if the probability of event occurrence rises to 1.0, it was found that the processing delay of HBT approaches a level similar to that of LEO under some high-density infrastructure conditions. Finally, in the 24.2 MB high-resolution image condition, when the number of infrastructures is large and the probability of event occurrence is high, a section where the processing delay of HBT exceeds the LEO is also observed. This suggests that if the LEO structure is low in resolution, it can maintain a high processing speed with a fixed amount of data, while HBT has a limitation of linearly increasing the amount of processing data due to the event-oriented structure. In this way, the interaction between resolution and structure shows that the selection of the optimal structure may vary depending on the monitoring environment and acts as a key factor to be considered when designing.

Event probability was also identified as a major variable that directly affects the processing delay of the HBT structure. In the low-event-occurrence-probability interval of 0.1 to 0.3, even if the number of infrastructures per LAP increases, the processing delay increases smoothly, which shows that the HBT structure can maximize system efficiency through an event-oriented lightweight processing method. In particular, this section suggests the possibility of maintaining an overwhelmingly lower processing delay than the LEO structure and providing stable performance even in a large infrastructure environment. On the other hand, when the probability of an event occurring increases from 0.7 to 1.0, the slope of the processing delay increases rapidly due to the increase in the number of infrastructures, and the calculation and transmission load of the HBT structure increases significantly. This reflects the structural characteristics that the throughput increases linearly in an environment where changes are frequent due to the limitation of the event-based structure. This trend means that there is an intersection in which the LEO structure records a lower processing delay under certain conditions, and it was observed in the experimental results that the location of this intersection varies depending on the resolution conditions. This intersection can provide a reference point for structure selection and shows that it is necessary to select an optimal structure that reflects the characteristics of the monitoring environment and load patterns when designing the system. Eventually, the HBT structure provides excellent efficiency at low event frequency, but the performance can converge or reverse with the LEO structure as the event frequency increases, so event probability acts as a key factor to consider in structural performance evaluation.

Quantitatively, taking the normalized delay of the direct LEO–terrestrial baseline as 100, Figure 4 shows that in the 132.5 MB ultra-high-resolution case the proposed HBT architecture keeps the normalized processing delay in the range of roughly 20–40 across all tested combinations of infrastructures per LAP and event probabilities, corresponding to about a 60–80% reduction compared to the baseline. In the 50 MB actual-resolution case, the delay of HBT increases with both the number of infrastructures and the event probability, but it still remains below about 60–70 even in the heaviest settings, which implies a delay reduction of roughly 30–40% relative to direct LEO–ground transmission. In the 24.2 MB high-resolution case, HBT continues to achieve normalized delays of around 20–80 under low-to-moderate event probabilities, whereas for the extreme combination of dense infrastructures and an event probability close to 1.0, the delay rises to approximately 120–130 and slightly exceeds the baseline plane at 100. These numerical trends confirm that the event-driven abstraction of the HBT structure yields substantial delay reductions in typical operating regimes, while also revealing the corner cases in which its advantage can diminish.

### 5.2. Measure of the Surveillance Resolution

The simulation was conducted in a two-dimensional grid environment, where a nomadic infrastructure moved along a consistent diagonal trajectory across the surveillance area. The movement pattern was deterministic, allowing the infrastructure to continuously traverse through various surveillance zones at a fixed direction and speed. In the HBT structure, surveillance coverage was provided by a grid of LAP nodes with predefined sensing radii and regular spatial intervals. In contrast, the satellite–LEO structure was modeled using multiple satellite nodes operating on a fixed visibility schedule and broader sensing radii. At each time step, the simulation evaluated whether the moving infrastructure was within the active surveillance coverage of each structure, and the SR was computed as the percentage of successfully tracked time steps throughout the entire simulation period. This configuration ensured a consistent and fair comparison of tracking performance across different system architectures under identical movement conditions.

In this experiment, we modeled the difficulty change in the monitoring environment by changing the moving speed of the nomadic infrastructure. The HBT structure is designed to provide high monitoring persistence based on fine-grained coverage and fast monitoring cycles of the LAP stage, and existing satellite–ground communication structures have a wider monitoring radius but relatively slow monitoring response. The faster you move, the more frequently you exceed the monitoring range, which makes the difference in the monitoring performance of each structure noticeable. Therefore, experimental configurations that change to speeds from 1 to 9 are meaningful in modeling the deterioration of the delayed environment in which the system must make tracking decisions within a shorter time period.

The proposed HBT structure is based on the basic design of HBT, in which one LEO manages nine HAPs and each HAP manages nine LAPs again. As a result, one LEO covers 81 LAPs, and the sensing radius between each layer is designed to satisfy the proportional relationship in area. However, in the actual environment, there is a possibility that the monitoring radius may be adjusted to a narrower or wider range than the theoretical radius, which can affect the tracking performance of the system. In order to reflect this situation, this experiment introduced the sensing radius of the LAP as a variable and quantitatively analyzed how the flexibility of the monitoring radius affects the probability resolution of the HBT structure. The sensing radius of the LAP was constructed by varying the values from 1 to 9, and the sensing radius of the LEO was fixed at 45 to match the ratio of 5, the median value of the LAP. Through this, we evaluated how fast the system could continuously monitor the moving infrastructure according to each radius value. Furthermore, it was possible to determine whether the tracking performance improved due to the expansion of the radius, as well as whether the saturation phenomenon or performance degradation of efficiency occurred above a certain radius. The purpose of this variable is to supplement the limitations of the static radius assumption when designing the monitoring system and to investigate the effect of adjusting the monitoring radius, which can be adapted to the dynamic environment.

**Figure 5:**
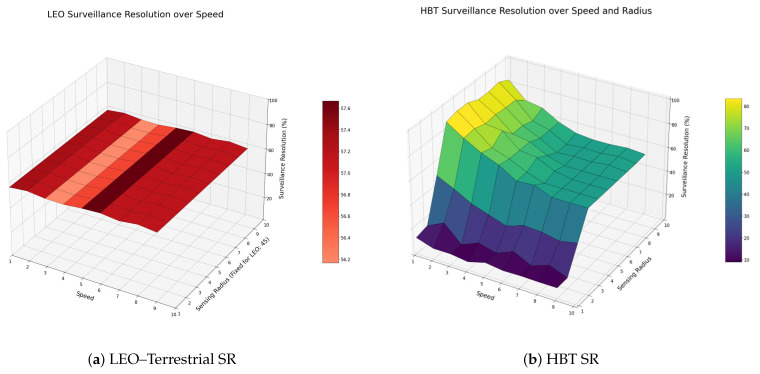
SR of the direct LEO–terrestrial baseline (**a**) and the proposed HBT architecture (**b**) as a function of infrastructure speed and sensing radius. A common color scale is used in both plots so that the SR gap is clearly visible even at intermediate speeds.

According to Figure 5, as the speed increased, the SR showed constant changes in both structures, but the trend was distinctly different for each structure. The HBT structure maintained a high SR of up to 88% or more in the sections from Speed 1 to 3, but the performance gradually decreased as the speed increased to 5 or more. In particular, at Speed 9, it fell to the 50% level, making it difficult to track fast-moving nomadic infrastructure in real time. This is due to the monitoring delay caused by the widening gap between the sensing period of the LAP and the actual location change. On the other hand, in the LEO structure, the SR remained stable in the range of about 56–58% over the entire speed section. It shows structural characteristics that are less sensitive to movement speed changes due to the characteristics of an LEO system with a wide monitoring radius and a fixed update period. In summary, HBT shows precise and excellent performance in a low-speed environment, but its performance decreases to a level similar to that of LEO in a high-speed environment, while LEO operates as a conservative monitoring structure that is resistant to speed changes while having low precision. As a result of measuring the SR performance by changing the sensing radius from 1 to 10, the overall probability resolution of the HBT structure improved significantly as the radius increased. At the level of 1 to 3, an overall low SR was shown due to the lack of coverage, and if the radius was 6 or more, more than 70% stable tracking was possible under most speed conditions. In particular, when the speed is less than the middle of 1 to 5, the radius expansion effect is maximized, and the surveillance performance is improved dramatically. This shows the strength that the HBT structure can flexibly adjust the system performance through the parameter of the sensing radius.

In addition, the experiments sweep key parameters such as the number of nomadic infrastructures per LAP (10–50) and the sensing radius (1–9) over a wide range. This parametric study effectively serves as a sensitivity analysis with respect to block density and coverage, and we observed that changing the fan-out between layers yields analogous trends; therefore, additional figures for alternative fan-out configurations are omitted for brevity.

## 6. Future Work

### 6.1. Weather-Aware Channel Modeling and Resilience Analysis

The present study intentionally adopts a simplified channel model in which each LEO–HAP and HAP–LAP link is represented by an effective average rate under clear-sky conditions. This abstraction keeps the focus on architectural differences between the direct LEO–terrestrial baseline and the proposed HBT hierarchy, but it does not explicitly capture weather-induced attenuation and outages, which can be significant for high-frequency LEO downlinks.

A natural extension is to incorporate weather-aware channel models into the delay and SR analysis. For example, each link can be associated with a weather state w(t) and a corresponding attenuation factor $\alpha_{l}\left(w\right)$ or outage probability $q_{l}\left(w\right)$. The effective link rate would then become $\alpha_{l}\left(w\right)R_{l}$, and the processing delay and SR metrics could be re-evaluated under time-varying weather conditions using stochastic or Monte-Carlo analysis. This would enable a quantitative comparison of the weather resilience of the direct LEO–terrestrial architecture and the HBT hierarchy.

We expect that explicitly modeling weather effects will further highlight the advantage of the proposed architecture. In adverse conditions, long LEO–terrestrial links at high carrier frequencies are more vulnerable to rain fade and cloud blockage, whereas short-range HAP–LAP links can be operated at more robust frequency bands and can exploit spatial diversity through neighboring LAPs and HAPs. Evaluating how the HBT framework can adapt its Infra Map updates, block assignments, and routing decisions according to the weather state is an important direction for future research.

### 6.2. HBT-Enhanced Hybrid Satellite–Aerial Architectures

The present study deliberately focuses on a direct LEO–terrestrial architecture as the quantitative baseline in order to isolate the impact of event-driven abstraction, block-level state management, and Infra Map updates on processing delay and SR. As discussed in Section 2, modern hybrid satellite–aerial systems such as LEO–UAV/HAP–ground networks primarily optimize physical connectivity and resource allocation while still forwarding raw sensing data or atomic task payloads across multiple hops. An important direction for future work is to embed the proposed HBT mechanisms into such hybrid architectures and perform a full system-level comparison.

Concretely, one can integrate the LAP/HAP-based abstraction and Infra Map management into representative hybrid topologies and jointly evaluate three classes of designs under identical scenarios: (i) a pure direct LEO–terrestrial baseline, (ii) conventional hybrid relays that forward full-resolution imagery or raw sensor streams, and (iii) HBT-enhanced hybrid architectures that propagate only delta updates. Extending both the analytical model and the simulation framework to these cases would clarify how much additional gain HBT can provide on top of existing hybrid connectivity and would quantify trade-offs between hop count, payload abstraction level, and tracking continuity in realistic space–air–ground integrated networks.

### 6.3. Security-Aware Interaction Protocols and System Integration

The present study assumes trusted links between the LEO, HAP, and LAP layers and does not explicitly model security mechanisms in order to focus on architectural aspects such as event-driven abstraction, block-level Infra Map management, and their impact on processing delay and SR. An important direction for future work is to incorporate data authenticity and confidentiality into this pipeline by introducing lightweight security mechanisms, for example message authentication codes (MACs) for delta updates and selective encryption for sensitive infrastructure state information. Such mechanisms will inevitably introduce computational overhead at LAPs and additional payload on the uplink, so re-evaluating processing delay, SR, and energy consumption under these more realistic security-aware conditions will be an essential next step.

A related extension is to study how the proposed HBT-based interaction model can be embedded into existing satellite platforms and protocol stacks that already implement their own security procedures and communication standards. Mapping event-driven abstraction messages and Infra Map updates onto the control or management planes of current LEO systems, and investigating representative integration scenarios, would make it possible to quantify the impact of security-aware protocols on signaling overhead and tracking performance. This, in turn, would provide a concrete path toward applying the proposed architecture in operational space–air–ground systems.

## 7. Conclusions

In this study, an HBT structure and an event-driven update method based on the Infra Map were proposed to overcome the limitations of real-time monitoring and network management in a highly mobile nomadic infra environment. Building on the architectural design in Section 4, the proposed LEO–HAP–LAP hierarchy manages nomadic infrastructures in block units and formalizes processing delay and SR as key performance metrics. This provides a unified framework for reasoning about how image size, event dynamics, and infrastructure density jointly affect both the signaling load and the tracking capability of satellite-based surveillance systems.

The evaluation results in Section 5 confirm that the proposed Hybrid Baseband Tracking (HBT) architecture provides concrete performance gains over the direct LEO–terrestrial baseline, quantitatively reducing the normalized processing delay from 100 to approximately 20–40 for the 132.5 MB ultra-high-resolution case and to below approximately 60–70 for the 50 MB actual-resolution case (a 30–80% delay reduction), achieving up to about 88% tracking continuity in SR (SR) experiments at low speeds, significantly higher than the baseline’s 56–58%, and maintaining more than 70% SR across most speed ranges when the Local Abstraction and Processing (LAP) sensing radius is set to six grid units or more. The HBT structure achieves substantially lower processing delay than the direct baseline when high or ultra-high image resolutions are used and the event probability is low to moderate because only compact abstraction payloads are transmitted instead of full images, though an intersection region appears where the delays become comparable as event probability and the number of infrastructures per LAP increase, highlighting event density as a critical design parameter; further SR analysis shows that HBT maintains much higher tracking continuity than the direct baseline at low and medium speeds, while its SR gradually converges toward the LEO value as speed increases, and that enlarging the LAP sensing radius beyond a certain threshold significantly improves SR across a wide range of mobility conditions.

In summary, these findings clarify both the scientific and practical contributions of the proposed approach. Scientifically, this paper establishes nomadic infra, HBT, and Infra Map-based abstraction as a general architectural basis for future space–air–ground networks and complements existing satellite-assisted IoRT and SAGIN schemes that have so far assumed simpler connectivity models. From a practical perspective, the results indicate that systems using large image payloads with moderate event rates are well suited to an HBT-like event-driven hierarchy that reduces delay and backbone load, whereas scenarios with extremely frequent events and relatively small images are better matched to hybrid designs that selectively fall back to direct LEO operation in the identified intersection region; moreover, the LAP deployment density and sensing radius should be dimensioned so that the resulting SR exceeds a target level for the expected speed range of nomadic infrastructures. Taken together, the proposed nomadic infra and HBT framework provides both a theoretical tool and concrete design guidelines for realizing efficient and scalable space–air–ground surveillance systems beyond conventional direct satellite–ground communication.

## Figures and Tables

**Table 1:** Comparison between direct LEO–terrestrial communication and HBT architecture.

Table		LEO–Terrestrial Communication	HBT Structure
**Latency**	**Propagation**	Similar delay based on same physical distance (up to 2.7 s)	
	**Processing**	High delay due to encoding/decoding and analysis of high-resolution images	Low delay due to string-based abstraction at LAP
**Transmission Volume**		Transmission of several MB per image or raw data	Transmission of lightweight abstracted data (tens to thousands of bytes)
**Queueing/Congestion**		High queuing delay due to large data size	Minimal queuing due to lightweight payloads
**Estimated Total Delay**		25–45 ms—higher due to heavy data processing and queuing caused by high transmission volume	10–30 ms—lower due to lightweight abstraction and minimal transmission overhead
**Data Processing Point**		Post-processing at satellite or ground station	Pre-processed at LAP and forwarded in abstracted form
**Real-time Capability**		Limited due to heavy data handling	High due to simplified and fast transmission
**Tracking Failure Risk**		High due to communication interference from atmospheric conditions and mobility of nomadic infrastructure	Low due to block-level relays and continuous location renewal
**Network Flexibility**		Low due to fixed satellite orbit paths	High due to redeployable and reconfigurable LAP/HAP

**Table 2:** Symbols and descriptions used in processing delay analysis.

Symbol	Description
*p*	Event occurrence probability per infra per slot
$N_{b}$	Average number of infrastructures per block
*B*	Number of blocks (under an LEO)
Δt	Slot duration
*m*	Merge factor at HAP (post-dedup fraction)
*r*	Rate limit/acceptance factor at HAP
$\lambda^{\mathrm{LEO}}$	Effective event arrival rate to LEO (after m,r)
$s_{\Delta }$	Delta payload size per event
$\Gamma^{\mathrm{LEO}}$	Offered traffic on HAP → LEO link
$s_{\mathrm{img}}$	Raw image payload size
$R_{\mathrm{LT}}$	LEO ↔ terrestrial link rate
$R_{\mathrm{HL}}$	LAP ↔ HAP link rate
$R_{\mathrm{LH}}$	HAP ↔ LEO link rate
$D_{\mathrm{LEO}}$	Transmission lower bound (direct baseline)
$D_{\mathrm{HBT}}$	Transmission lower bound (HBT, two hops)

**Table 3:** Symbols and descriptions used in the SR section.

Symbol	Description
$d_{i}\left(t\right)$	Distance at time *t* between infrastructure *i*
	and the nearest covering platform
*R*	Coverage radius/threshold used for success criterion ($d_{i}\left(t\right)\leq R$)
${\mathrm{cov}}_{i}\left(t\right)$	Coverage flag at time *t*; 1 if $d_{i}\left(t\right)\leq R$, otherwise 0
*T*	Number of evaluation time slots in the window
${\mathrm{SR}}_{i}$	Per-infrastructure SR: $\frac{1}{T}\sum_{t=1}^{T}{\mathrm{cov}}_{i}\left(t\right)$
I	Set of infrastructures under evaluation
|I|	Cardinality of I (number of infrastructures)
SR	System-level SR: $\frac{1}{\left|I\right|}\sum_{i\in I}{\mathrm{SR}}_{i}$

## Data Availability

Data sharing The original contributions presented in this study are included in the article.

## Abbreviations

The following abbreviations are used in this manuscript:

HBT	Hierarchical Block Tracking
LEO	Low Earth Orbit
HAP	High-altitude Platform
LAP	Low-altitude Platform
GEO	Geostationary Orbit
RTT	Round-Trip Time
SR	Surveillance Resolution
